# Atomic Study on Tension Behaviors of Sub-10 nm NanoPolycrystalline Cu–Ta Alloy

**DOI:** 10.3390/ma12233913

**Published:** 2019-11-27

**Authors:** Weibing Li, Xiao Wang, Libo Gao, Yang Lu, Weidong Wang

**Affiliations:** 1 ZNDY of Ministerial Key Laboratory, Nanjing University of Science and Technology, Nanjing 210094, China; njustlwb@163.com; 2 School of Mechano-Electronic Engineering, Xidian University, Xi’an 710071, China; wangxiao9626@outlook.com (X.W.); lbgao@xidian.edu.cn (L.G.); 3 Research Center of Micro-Nano Center, Xidian University, Xi’an 710071, China; 4 CityU-Xidian Joint Laboratory of Micro/Nano-Manufacturing, Shenzhen 518057, China; yanglu@cityu.edu.hk; 5 Department of Mechanical Engineering, City University of Hong Kong, Kowloon 999077, Hong Kong

**Keywords:** Cu–Ta alloy, nanopolycrystalline, tension behaviors, molecular dynamics simulation

## Abstract

Atomic simulations give a good explanation of the changes in the physical properties of a material. In this work, the tension behaviors of nanopolycrystalline Cu–Ta alloys are investigated through molecular dynamics (MD) simulations, and the influences of several important factors on the mechanical properties of the materials are studied. Firstly, nanopolycrystalline Cu–Ta (10 at %) alloy models with sub-10 nm grains are established by using the method of replacing the grain boundary atoms. Then, the effects of temperature, pressure, and strain rate on the mechanical properties of nanopolycrystalline Cu–Ta alloy are studied, and the elastic modulus and flow strength are obtained. The observations from the simulation results show that the elastic modulus and flow strength increase with the increasing of grain size for sub-10 nm nanopolycrystalline Cu–Ta alloys, and the elastic modulus increases firstly and then stabilizes as the strain rate increases. Finally, according to the evolution of dislocations and twin crystals, the plastic deformation mechanism of nanopolycrystalline Cu–Ta alloy during the stretching process is discussed in depth.

## 1. Introduction

Tantalum (Ta) and tantalum alloy are ideal materials for many fields due to their excellent properties such as high melting points, corrosion resistance, and excellent phase stability. Many researchers have verified these excellent properties through experimental and theoretical studies [1,2,3,4,5,6,7]. Dewaele et al. [1] studied the effects of pressure on the yield strength of Ta in a diamond anvil cell (DAC) up to 93 GPa, and the DAC experiments also demonstrated that the body centered cubic (BCC) structure remained stable up to 135 GPa. Wu et al. [5] investigated the elastic and thermodynamic properties of Ta at high pressure up to 350 GPa. Liu et al. [6] used the extended Finnis–Sinclair (EFS) potential to study the melting properties of tantalum under high pressure and calculated the melting point of tantalum under different pressures. Li et al. [7] simulated the tensile test of single-crystal Ta via the molecular dynamics (MD) method and studied the influence of temperature and pressure on the elastic modulus in the <100> direction of single-crystal Ta.

Because the tantalum in the Cu–Ta system is a high-melting-point metal, the melting point of copper is relatively low. The effective combination of the two makes the Cu–Ta alloy exhibit excellent mechanical strength and structural stability under high temperature. Therefore, in addition to the previous work, many studies have been carried out on the deformation mechanism of the Cu–Ta alloy [8,9,10,11,12]. Zeng et al. [8] measured the elastic modulus and hardness of the Cu–Ta multilayer film using the nanoindentation test. Zhu et al. [9] studied the tensile deformation of the Cu–Ta multilayer film. It was found that the grain boundary of the deformation region near the channel crack of the top copper layer was arranged in a row. Wang et al. [10,11] studied the shear band deformation of Cu–Ta nanomultilayer films and considered the strain rate sensitivity, which confirmed that the influence of dislocations and grain boundary shift on plastic deformation is mainly dependent on the strain rate and grain size. Frolov et al. [12] studied the effect of Ta on the grain growth and mechanical strength of nanopolycrystalline Cu–Ta alloy based on MD theory. A Cu–10 at % Ta alloy was produced by ball milling with a structure composed of Cu and Ta grains less than 10 nm in diameter. Therefore, we will focus on this Cu–Ta alloy in this study.

Although there have been a lot of studies [13,14,15,16,17,18,19,20,21] on preparation methods and deformation mechanisms of the Cu–Ta alloy, few researchers pay any attention to analyzing some main factors influencing the mechanical properties of the Cu–Ta alloy quantitatively and systematically. According to Frolov’s work [12], the main purpose of this study is to quantitatively analyze the influence of grain size and strain rate on the mechanical properties of Cu–Ta (10 at %) alloy by molecular dynamics method and verify the rationality of the method of establishing the Cu–Ta alloy model.

## 2. Materials and Methods

### 2.1. Physical Modeling

Establishing the correct simulation model is the basis and premise for carrying out MD simulations. It is very important to build the simulated models accurately according to the topology of polycrystalline materials, taking into consideration research objectives and time consumption. In this paper, the Voronoi method [22] was used to establish nanopolycrystalline Cu–Ta alloy models.

**Figure 1:**
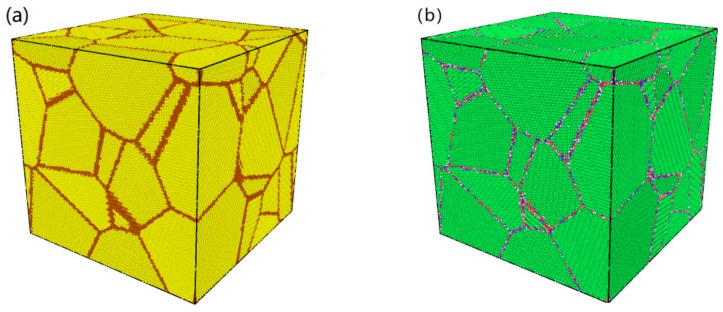
Physical models of nanopolycrystalline Cu and Cu–Ta alloys. (**a**) Sketch map of nanopolycrystalline Cu cubic with a grain size of 10 nm. The golden and brown atoms indicate Cu atoms in grains and grain boundaries, respectively. (**b**) Lattice map of Cu–Ta alloy colored by polyhedral template matching (PTM). The blue is body centered cubic (BCC), green is faced centered cubic (FCC), red is hexagonal close packed (HCP), yellow is incommensurate case (ICO), and white is other lattice structures.

In this study, the method of replacing the grain boundary atoms (RGBA) was used to build the nanopolycrystalline Cu–Ta alloy models. First, a polycrystalline copper cubic was established with a side length of 30 nm and grain size of 10 nm so that its grain number was 27. This cubic model had a total of 2,295,585 Cu atoms, among which 275,121 Cu atoms were in the grain boundaries marked in brown as shown in Figure 1a. Then, 229,558 Cu atoms in the grain boundaries were randomly selected to be replaced by Ta atoms for the purpose of establishing a Cu–Ta (10 at %) alloy model, i.e., RGBA model. Figure 1b shows the physical model of the nanopolycrystalline Cu–Ta alloy and its lattice map colored by the polyhedral template matching (PTM) algorithm. The above processes were performed in Ovito (post-processing visualization software, version 2.9.0, Alexander Stukowski, Wiesbaden, Germany) with the help of MATLAB.

### 2.2. Methodology of Atomistic Simulations

MD simulations in this paper were performed using the large-scale atomic/molecular massively parallel simulator (LAMMPS, version 31, Mar17, Sandia National Laboratories, California, America) [23]. During the process of MD simulations, the classical embedded-atom method (EAM) potential was used to describe the interactions of both Cu–Cu and Ta–Ta [7]. In this study, the angular-dependent interatomic potential (ADP) [24] was chosen to describe the interaction between Cu and Ta atoms. In the ADP potential, the total energy of the system is calculated by the following equation

(1) $$E_{tot}=\frac{1}{2}\sum_{i,j\left(i\neq j\right)}\Phi_{S_{i}S_{j}}\left(r_{ij}\right)+\sum_{i}F_{S_{i}}\left({\bar{\rho }}_{i}\right)+\frac{1}{2}\sum_{i,\alpha }{\left(\mu_{i}^{\alpha }\right)}^{2}+\frac{1}{2}\sum_{i,\alpha ,\beta }{\left(\lambda_{i}^{\alpha \beta }\right)}^{2}-\frac{1}{6}\sum_{i}V_{i}^{2}$$

where the subscripts of *i* and *j* enumerate atoms and the superscripts *α*, *β* = 1, 2, 3 represent the Cartesian components of vectors and tensors. $\Phi_{S_{i}S_{j}}\left(r_{ij}\right)$ is the pair interaction energy between atom *i* and atom *j*. The second term is the energy of embedding an atom of chemical species $S_{i}$ in the host electron density ${\bar{\rho }}_{i}$ induced at site *i* by all other atoms. The remaining terms represent the noncentral character of bonding, including the dipole vectors and quadrupole tensors.

During the MD simulations, the periodic boundary conditions (PBC) were used in three directions of the model. The time step was set as 1 fs. The Cu–Ta system was first relaxed about 50 ps under the canonical ensemble (NVT) in order to achieve the minimum energy and stable structures. The effect of specific hydrostatic pressure ranging from 0 to 140 GPa on the elastic properties of the Cu–Ta alloy was then ensured under the isothermal–isobaric ensemble (NPT) [25]. Finally, the tension loading ranges from 5 × 10^8^ s^−1^ to 4 × 10^9^ s^−1^ were applied to the x-direction of the Cu–Ta cubic [26,27].

## 3. Results and Discussion

### 3.1. Dependence of Grain Size

A nanopolycrystalline Cu–Ta alloy model was established, and its sketch map with an average grain size of 10 nm was given, as mentioned above. The potential function of the ADP type was selected. In order to investigate the effect of grain size on the tensile properties, the plastic deformation mechanism, and the strain rate effect of polycrystalline Cu–Ta alloy at nanoscale, the same method of RGBA was utilized to establish various cubic models with the same grain number of 27 but different model sides. The RGBA models of Cu–Ta alloy have different side lengths of 6, 9, 12, 15, 18, 21, 24, 27, and 30 nm, with corresponding average grain size of 2, 3, 4, 5, 6, 7, 8, 9, and 10 nm, and their atom numbers range from 18,382 to 2,295,585. It should be pointed out that all of the RGBA models have the same Ta atom percentage of 10%. Based on the PTM algorithm, the atomic compositions of the RGBA models were analyzed, and the lattice distribution obtained is shown in Figure 1b. Among the lattice structures, the blue is BCC lattices, the green is FCC lattices, the red is HCP lattices, the yellow is ICO lattices, and the white is other lattices, respectively. It can be seen that the size and shape of the grains in the model are random. In the RGBA model, the internal atoms of the grains are all FCC lattices, and the atoms at the grain boundaries have various lattice types.

**Figure 2:**
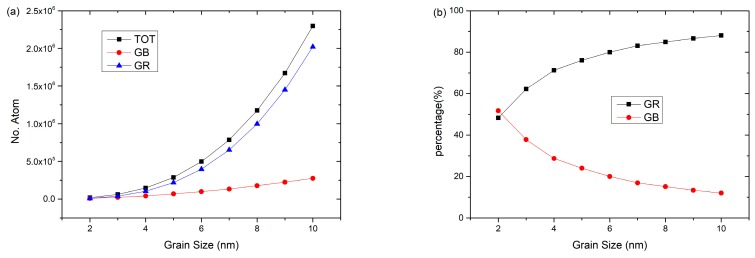
The atom numbers of total atoms (TOT), atoms in grains (GR), and atoms in grain boundaries (GB) as a function of grain size. (**a**) The atom numbers of grains and grain boundaries vs. the average grain size. (**b**) The atom proportion of GR and GB vs. the average grain size.

A large number of studies [28,29,30,31,32,33] have shown that the internal structures and grain boundary density of nanopolycrystalline materials have great influences on their mechanical properties and behaviors, especially deformation mechanisms. Combined with dislocation analysis (DXA) methods, the numbers of total atoms (TOT), atoms in grains (GR), and atoms in grain boundaries (GB) of each RGBA model are calculated to explore the internal atomic composition and grain boundary density of the Cu–Ta alloy models with different grain sizes. Figure 2 shows the atom numbers of TOT, GR, and GB as a function of grain size.

It can be seen from Figure 2a that as the grain size increased, the atom numbers of TOT, GR, and GB increased and TOT and GR increased greatly. As shown in Figure 2b, for the case of 2 nm grain size, the atom number of GB occupied the largest proportion among all the RGBA models. However, as the average grain size increased, the atom proportion of GR increased rapidly, so that the atom proportion of GB decreased rapidly. It is obvious that the atom proportion of GB was smaller than that of GR except for the case of 2 nm grain size, and the gap between GR and GB got larger and larger as the grain size increased; that is to say, the internal atomic composition of the model changed greatly. It should be noted that the RGBA models used in this study had the same number of crystal grains, i.e., 27.

**Figure 3:**
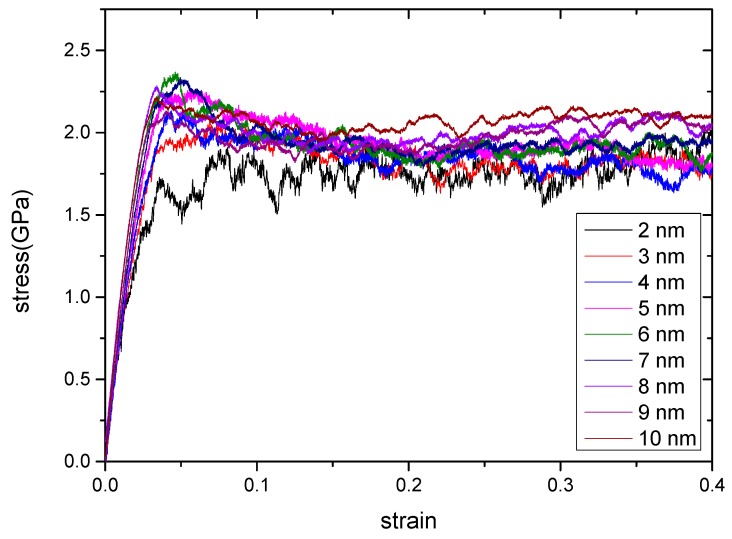
The stress–strain curves obtained by stretching Cu–Ta alloy models with different grain sizes at a temperature of 300 K and a strain rate of 5 × 10^8^ s^−1^.

In the MD study, the ambient temperature was set as 300 K and the strain rate as × 10^8^ s^−1^. First, uniaxial tension simulations were performed for all the RGBA models with grain sizes ranging from 2 to 10 nm. The uniform strain was still applied on the *x*-axis, and the y- and z-axes remained freely contracted. The stress–strain curves were obtained by stretching Cu–Ta alloy models with different grain sizes as shown in Figure 3.

It can be concluded from Figure 3 that the stress–strain curves obtained by stretching Cu–Ta alloys with different grain sizes have the same general trend. All the curves in the figure have a linear relationship between stress and strain at the beginning, which means that Cu–Ta alloys with different grain sizes first undergo the elastic phase. At this time, the elastic constant of the corresponding size can be obtained by fitting the initial linear phase. When the strain increases to a certain value, the material reaches the yield point, and then, the RGBA begins to yield and plastically deforms. It is worth noting that although the RGBA model in the yield stage undergoes plastic deformation, it still undergoes elastic deformation at the same time, and the slope of the curve is smaller than the slope of the initial elastic phase. As the strain continues to increase, the stress reaches a maximum, namely, the peak stress. After that, the stress continues to drop and eventually reaches a relatively stable state. At this time, the stress is in an oscillating state, and the RGBA model in the process is always in plastic deformation. Generally, the flow strength describes the strength of the plastic deformation of materials. The flow strengths are obtained by fitting the average value of the stress between the strain of 0.2 and the strain of 0.4. Table 1 gives the elastic modulus and the flow strength of nanopolycrystalline Cu–Ta alloys with different grain sizes.

From Table 1, it can be found that both the elastic modulus and the flow strength increase with the increasing of grain size; moreover, the elastic modulus varies more significantly than the flow strength.

### 3.2. Dependence of Strain Rate

**Figure 4:**
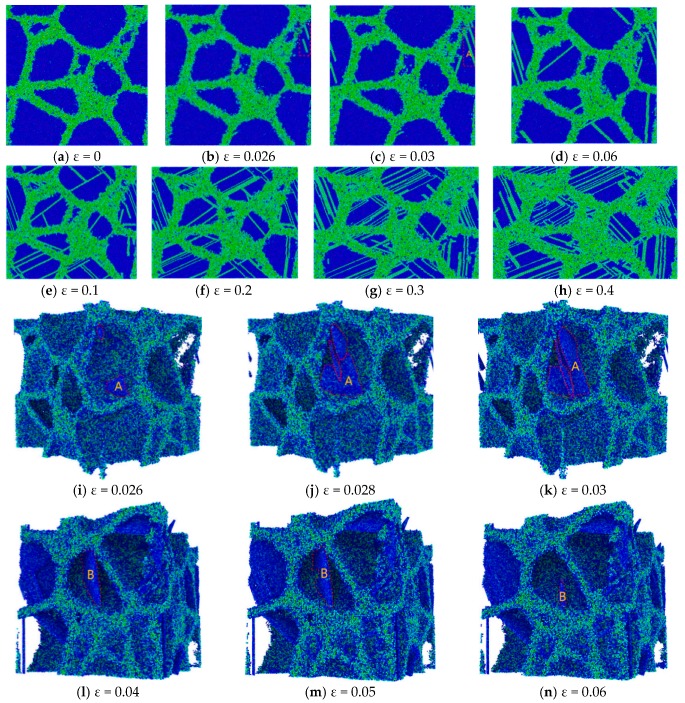
Defect activities in RGBA models with a grain size of 10 nm at various ε. (**a**–**h**): snapshots of atomic configurations at different strains; (**i**–**n**) evolution of grain boundaries and dislocations during tension, with atoms colored by the centro-symmetry parameter (CSP).

Firstly, let us focus on analyzing the atomic configuration of the RGBA model with an average grain size of 10 nm during its stretching process. Figure 4a gives the initial atom configuration of the RGBA model after sufficient relaxation at a strain of *ε* = 0. The observations from MD simulations show that the Cu–Ta alloy presents an elastic deformation when the strain lies between 0 and 0.026, which can be verified from the stress–strain curve in Figure 3. It is indeed found from Figure 4a,b that there are no dislocations and crystal twins introduced in the RGBA model, only the crystal lattice inside the grains. According to Figure 3, it can be found that the Cu–Ta alloy reaches the yield point at *ε* = 0.026 and then steps into its yielding stage showing plastic deformation. Comparing Figure 4c with Figure 4b, it is obvious that the dislocations show an expansion process. In order to clearly observe the expansion of dislocation-A shown in Figure 4c further, the centro-symmetry parameter (CSP) algorithm was utilized to demonstrate its growth in Figure 4i–k. As the strain steps from 0.026 to 0.03, the dislocations grow rapidly.

At *ε* = 0.03, the dislocations extend to the grain boundaries of the original grain and begin to grow slowly until they stop. In addition, focusing on the motion of dislocation-B, a complete crystal plane is gradually formed while ε lies between 0.04 and 0.05. The crystal face just formed is gradually destroyed with the increase of strain due to its unstable structure under external loading. At this point, the strain reached 0.06, and Figure 4l–n demonstrates this process. Thereafter, as the strain is further increased, Fig.4 d–h show that there is a plurality of deformed twin nucleation at many crystal faces, which respectively expand to form a twin plane in the opposite grain boundary direction. Comparing a few figures shows that there is a lot of dislocation motion. New dislocations emerge from the interior of the grain, while some dislocations migrate and accumulate at the grain boundaries, and new dislocations repeat the process. In addition to the phenomenon of deformation twinning and twin boundary migration, as well as dislocation motion, grain boundary slip can also be observed.

Statistically speaking, dislocations and crystal twins are more likely to occur at large-angle grain boundaries, and the directions produced are mostly perpendicular to the stretching direction or at an acute angle to the stretching direction. With the growth of crystal twins, annihilation occurs at the original grain boundaries, leaving new twin boundaries, which in turn form new crystal faces, and new grains are produced. These above phenomena are consistent with previous studies [28,34,35,36,37,38].

**Figure 5:**
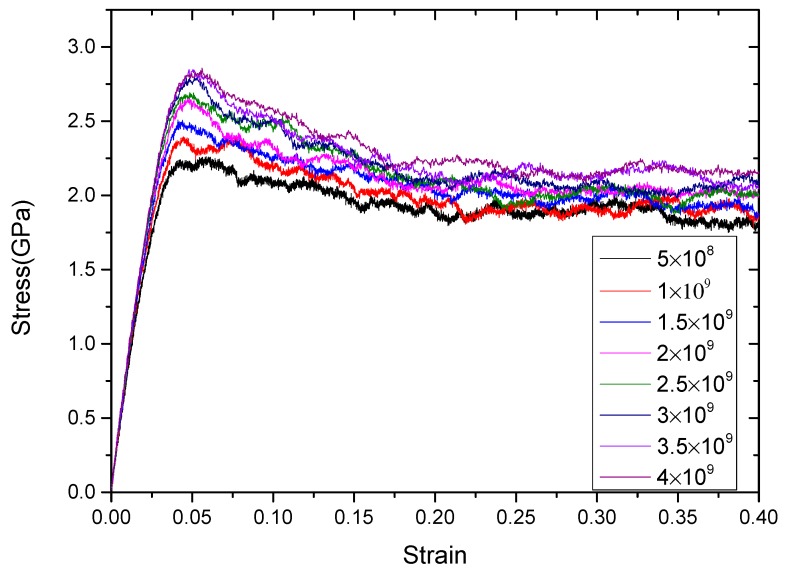
The stress–strain curves obtained by stretching 5 nm grain sizes at a temperature of 300 K and different strain rates.

After clarifying the changes of grain and grain boundaries during the stretching process, the influences of strain rate on the mechanical properties of RGBA models are concerned in the following section. In order to study the effect of strain rate on the elastic modulus of RGBA, uniaxial tension simulations were carried out at different strain rates for RGBA models with grain sizes of 5 nm and 10 nm, respectively. The strain rate ranged from 5 × 10^8^ s^−1^ to 4 × 10^9^ s^−1^, and the calculated stress–strain curve is shown in Figure 5.

In Figure 5, the slope of the curve of the elastic phase increased with the increase of the strain rate, whether it is the 5 nm or 10 nm RGBA model. Table 2 shows the elastic modulus and flow strength of the 5 nm and 10 nm RGBA models at different strain rates. It can be seen that when the strain rate changed from 5 × 10^8^ s^−1^ to 2.5 × 10^9^ s^−1^, the elastic modulus continuously increased. However, when the strain rate was larger than 2.5 × 10^9^ s^−1^, the elastic modulus kept a relatively stable value. The elastic modulus characterizes the bonding force between the atoms in the elastic stage; therefore, the strain rate has a greater influence on the plasticity of the material. The elastic modulus of the 5 nm RGBA model no longer changed with the strain rate when it reached about 78 GPa. The elastic modulus of the 10 nm RGBA model was stable at around 86 GPa.

The yield strength and flow strength are influenced by the strain rate, which may be related to the stress relaxation during stretching. It is assumed that the total deformation remains unchanged at a certain moment in the stretching process, and the elastic deformation is continuously converted into plastic deformation so that the stress is continuously reduced. However, stress relaxation requires a time process. The faster the stretching speed, the less obvious the stress relaxation phenomenon. The macroscopic performance is the increase of the flow strength, while the corresponding value of the yield strength is higher. Moreover, at high strain rates, twins appear, which are also responsible for the increase of flow strength with strain rate.

## 4. Conclusions

In this paper, nanopolycrystalline Cu–Ta alloy models were established by using the RGBA method, i.e., replacing the grain boundary atoms. Via MD simulating the stretching process of the Cu–Ta alloy and comparing the experimental results, it was found that the RGBA models were generally in line with the actual situation. Then, the tension behaviors of RGBA models with different grain sizes were further studied in this work. From the MD simulation results, it can be concluded that the elastic modulus increases as the grain size increases, which can be attributed to the decrease of the atom percentage of the grain boundary; this is because, in its essence, the elastic modulus is the binding force between the atoms in the elastic phase. Finally, the effect of strain rate on the mechanical properties of the Cu–Ta alloy was analyzed. The observations from the MD simulation results show that the elastic modulus first increases with increasing strain rate and then tends to a constant value, and the flow strength increases as the strain rate increases. In addition, the distribution of dislocations within the grains at different strain rates were calculated, and the plastic deformation mechanisms were analyzed in this paper. According to the MD simulation results, it also can be concluded that the strain rate has a more significant effect on the plastic properties of the nanopolycrystalline Cu–Ta alloy. These results provide useful knowledge for preparing better Cu–Ta alloys in the future.

## Figures and Tables

**Table 1:** The elastic modulus and flow strength of nanopolycrystalline Cu–Ta alloys.

Grain Size (nm)	Elastic Modulus (GPa)	Flow Strength (GPa)
2	48.26	1.78
3	61.92	1.79
4	68.86	1.81
5	69.73	1.87
6	75.76	1.89
7	76.13	1.91
8	80.44	2.01
9	81.53	2.03
10	85.44	2.09

**Table 2:** The elastic modulus and flow strength of the 5 nm and 10 nm RGBA models.

Strain Rate (s^−1^)	5 nm		10 nm	
	Elastic Modulus (GPa)	Flow Strength (GPa)	Elastic Modulus (GPa)	Flow Strength (GPa)
5.0 × 10^8^	68.38	1.87	75.98	2.09
1.0 × 10^9^	72.71	1.91	80.26	2.16
1.5 × 10^9^	74.17	1.97	81.64	2.21
2.0 × 10^9^	77.97	2.04	85.37	2.23
2.5 × 10^9^	78.43	2.05	86.72	2.27
3.0 × 10^9^	78.88	2.07	86.54	2.31
3.5 × 10^9^	78.55	2.13	86.33	2.34
4.0 × 10^9^	78.46	2.17	86.64	2.36
